# Botulism in Wild Birds and Changes in Environmental Habitat: A Relationship to be Considered

**DOI:** 10.3390/ani9121034

**Published:** 2019-11-26

**Authors:** Elena Circella, Antonio Camarda, Luca Bano, Giacomo Marzano, Roberto Lombardi, Francesco D’Onghia, Grazia Greco

**Affiliations:** 1 Department of Veterinary Medicine, University of Bari "Aldo Moro", s.p. Casamassima km 3, 70010 Valenzano BA, Italy; antonio.camarda@uniba.it (A.C.); roberto.lombardi.vet@gmail.com (R.L.); francescopaolo.donghia@uniba.it (F.D.); grazia.greco@uniba.it (G.G.); 2 Istituto Zooprofilattico Sperimentale delle Venezie, 31100 Treviso, Italy; lbano@izsvenezie.it; 3 Wildlife Nature Reserve “Torre Guaceto”, BR 72012 Carovigno, Italy; giacomomarzano@gmail.com

**Keywords:** wildlife conservation, human activities, nature reserve, botulism, wetland

## Abstract

**Simple summary:**

Human activities, even those aimed at improving a natural area, can interfere with wildlife and their environment, potentially leading to some changes. In this paper, a botulism outbreak which occurred in waterfowls in a nature reserve after a conservative action is reported. In particular, an artificial pond was created in order to improve the environment of waterfowls, but gray mullets (Mugil cephalus) settled in the pond and proliferated. Fish mortality was observed during summer, leading to an accumulation of decaying organic material, thus creating the optimal conditions for Clostridium botulin growth and toxin production. In the same period, the botulism outbreak with flaccid paralysis and sudden mortality rapidly occurred in waterfowls. The toxin mosaic type C/D was identified as responsible for the disease outbreak. The outbreak rapidly resolved after the removal of the fish carcasses, highlighting the importance of a correct management for any action in natural contexts. In conclusion, before considering any activity in wildlife habitats, it is important to assess first its possible impact on wildlife.

**Abstract:**

Any human activity, even if aimed at the improvement of a natural area, can potentially affect wildlife, leading to possible short-term or long-term changes due to the human–wildlife interaction. In this study, a botulism outbreak which occurred in waterfowl in a nature reserve after a conservative environmental action is reported. More than 180 different species of wild birds, including seventy waterfowl species, live in the area. The wildlife reserve rangers built an artificial pond equipped with draining canals in the wetland in order to improve the environment of waterfowl species and to facilitate their supply of food. Then, presumably due to tidal rides, gray mullets (*Mugil cephalus*) arrived from the sea and settled in the pond. The number of fishes gradually increased, and several fishes died with a peak of mortality in the summer of 2017, creating a great amount of decaying organic material and the optimal conditions for *Clostridium botulinum* growth and toxin production. A botulism outbreak then occurred rapidly and was characterised by flaccid paralysis and sudden mortality of the birds. Seven mallard ducks (*Anas platyrhynchos*), 4 common teals (*Anas crecca*), 1 garganey (*Anas querquedula*), 2 wood sandpipers (*Tringa glareola*), 1 little egret (*Egretta garzetta*), 1 little grebe (*Tachybaptus ruficollis*), and 4 Eurasian coots (*Fulica atra*) were found dead. Interestingly, the toxin identified as responsible for the disease outbreak was the mosaic of type C and D toxins (C/D type). The prompt removal of the fish carcasses led to a rapid resolution of the outbreak of the disease, highlighting the relevance of a correct management for any action in environmental contexts. The conclusion is that any human activity in wildlife habitats should be carefully considered in order to assess the possible impacts and to quickly identify the possible risks of changes in wildlife population.

## 1. Introduction

Interest for wildlife conservation is increased in public opinion, and it is already known that human activities can have different effects on wildlife and their environment. Some activities such as industries and road and building constructions have a great direct impact because of the heavy changes on the habitat and the reduction of geographical areas inhabited by wild species [1]. Other actions may have less evident effects that, however, can lead to interferences on wildlife. For example, even activities considered as eco-friendly and noninvasive for wildlife such as hiking, biking, and birdwatching in natural areas may create bother and noise, leading wild animals to run away and hide [2,3,4,5].

Any action of focused interventions for improving the condition of a natural area can potentially have negative effects on wildlife, leading to short-term or long-term changes in the environment due to human–wildlife interaction. For instance, supplementary feeding is used for attracting animals to tourists because it can facilitate close observation and interaction with wildlife. This can however result in negative effects on wildlife [6], since long-term provision of food can result in a dependency of animals on humans or in their getting used to human contact with reduction of instinctive fear [7]. Sometimes, risks of aggression for humans also occur due to animals seeking food [6], although individual variation in selection behaviour with respect to supplementary feeding has been observed [8].

Supplementary feeding is used also in wildlife management to aid wild species, particularly in winter or in conservation programs of declining or endangered species [6] In avian species, effects of supplementary feeding may have positive impacts on the survival of young birds in the reproduction season [9,10], increased growth rates of chicks [10], reduction in aggression related to fight for food among chicks [11], and improvements in fledging [12].

Nevertheless, supplementary feeding leads also to earlier laying dates one week or, in some cases, as long as one month depending on frequency and type of food [10], creating a possible imbalance among wild species. Likewise, the increase of the number of birds around feeders can lead to indirect ecological effects due to higher animal density and competition, with a possible increase of bird mortality around the feeders [13]. An increased risk in disease transmission can occur, as observed for Salmonella [10], *Mycoplasma gallisepticum* [14], and *Trichomonas* infection [15]. Another possible problem related to supplementary feeding could be the persistence of food in the environment that could also lead to the replication of *Clostridium botulinum (C. botulinum)* and the production of its toxin, particularly frequent in the presence of decomposing organic material [16], with consequent outbreak of botulism.

Botulism is a severe paralytic disease caused by botulinum neurotoxins (BoNTs) produced by *C. botulinum,* anaerobic, spore-forming, Gram-positive bacterium belonging to the genus *Clostridium. C. botulinum* is found in soils and the intestinal tract of animals, but it may be particularly frequent in water environment. Especially in the presence of decaying organic material, *C. botulinum* replicates by releasing the toxins.

BoNTs are classified into 7 serotypes (A–F). Avian botulism was observed in waterfowls in 1910 for the first time [17], and type C toxin was later identified as responsible for the disease. Mallard ducks (*Anas platyrhynchos*) and teals (*Anas crecca*) as filter feeding and dabbling waterfowls are among the species at higher risk for contracting botulism [18]. Since 1917, botulism was also reported in chickens, turkeys, pheasants, ducks, and peafowls [19]. Clinical signs of botulism are similar both in domestic fowls and wild birds and consist of flaccid paralysis of neck, wings, and eyelids [17,19]. Clinical signs and mortality occur within hours or 1–2 days depending on the toxin doses [20,21].

Type C, particularly, and D also are responsible for most cases of avian botulism [22,23]. Several outbreaks of type C botulism in wild species have been reported in several countries of Europe such as Denmark, Norway, the Netherlands, Hungary, the Czech Republic, Serbia, Slovenia, France, Germany, Ireland, the United Kingdom, and Spain [24]. Type C botulism was also reported in northern Italy [25]. Recently, some cases of botulism in waterfowl in Europe and Japan caused by a mosaic of type C and D toxins (C/D type) were reported and BoNT C/D is currently considered the main type involved in avian botulism outbreaks [16,26,27].

This study describes a botulism outbreak due to the exposure to BoNTs type C/D that occurred in waterfowls after a conservative action carried out in a wildlife reserve.

## 2. Materials and Methods

The botulism outbreak occurred in a wildlife preservation area named *Torre Guaceto* (40°42’54.69” N, 17°47’59.58” E), which, since 1981, is recognised as a “wetland of international interest” from the Ministry of Agriculture and Forestry of Italy. *Torre Guaceto* is included in the *Specially Protected Areas of Mediterranean Importance (SPAMI)* since 2008 and has an extension of 1500 hectares (ha) with a wetland of 200 ha, a Mediterranean scrub of 70 ha, a coast of about 8 km in length, and an area of 800 ha extensively cultivated with olive grove and wheat. More than 180 different migratory and nonmigratory species of wild birds, with 77 waterfowl species, live in the area. The list of waterfowl species of the reserve is reported in Table 1 (census performed according to References [28,29]).

An artificial pond equipped with draining canals was built in the wetland in order to improve the environment of waterfowl species and to facilitate their supply of food. Nevertheless, probably due to tidal rides, gray mullets (*Mugil cephalus)* arrived from the sea and settled in the pond. Their number gradually increased, and several of them died with a peak of mortality recorded in the summer of 2017 when daytime temperatures were in the mid-30s (°C). In the same period, many birds were found dead. Within a few hours after their finding, the reserve rangers sent them to the Avian Diseases Unit of the Department of Veterinary Medicine of University of Bari (Italy) for necropsy and laboratory investigations. Tissue samples (liver, gut, brain, and blood clots from the hearts) were rapidly collected and stored at –20 °C for diagnostic assays performed in the Avian Diseases Unit and completed in the Istituto Zooprofilattico Sperimentale delle Venezie (Italy).

As a possible etiological agent of neurological disorders in wild birds, brain samples were screened for the presence of Newcastle disease and influenza A viruses according to previously described methods [30]. In any case, anamnestic data and clinical signs led to a presumptive diagnosis of botulism. Therefore, one and half grams of each sample of liver and intestinal content were mixed with saline solution (vol/vol) and added to 12 mL of fortified cooked meat medium (FCMM) [31].

The inoculated tubes and the washing solutions of the samples were immersed in a hot bath (71 °C) for 10 min, cooled in water, and incubated at 37 °C in a Bactron IV anaerobic chamber (Shel Lab, Cornelius, OR, USA). After 48 h of incubation, 175 µL of each broth culture was collected from the bottom of the tubes and DNA was automatically extracted (Microlab Starlet, Hamilton, Bonaduz, Switzerland) using a MagMax Total Nucleic Acid Isolation kit (Ambion/Life Technologies, Carsland, CA, USA). PCR protocols for *C. botulinum* neurotoxin genes types A to F were applied in accordance with previously published conditions [32]. Type of mosaic neurotoxin gene was assessed by biomolecular techniques, as described elsewhere [26]. PCR-positive FCMM broth cultures were plated on egg yolk medium (EYA) produced with Blood Agar Base No.2 (BAB2) (Oxoid, Hampshire, United Kingdom) as a base and 50 mL/L of a yolk solution composed of 25 mL of fresh yolk and 25 mL of saline solution. A further 50 µL of FCMM was collected from the bottom of the tubes and plated on BAB2 with 5% defibrinated sheep blood and with agarose content enhanced to 2.5% to contain the spread of swarming clostridia (e.g., *Clostridium sporogenes*) on the agar surface. The EYA and BAB2 plates were incubated for 48 h in an anaerobic cabinet (Shel Lab) with an atmosphere composed of 5% hydrogen, 5% carbon dioxide, and 90% nitrogen. Suspected colonies observed in the EYA (lecithinase and small lipase positive colonies) and BAB2 (weakly hemolytic small colonies) were collected and transferred to a further 10 mL of FCMM in tubes. The broth cultures were incubated in anaerobic conditions at 37 °C for 48 h and tested by PCR protocols for toxin types A–F as described above. Supernatants of PCR-positive broth cultures were filtered with a 0.45-mm pore-size filter (Millipore, Tullagreen, Ireland) and tested for BoNTs by the Mouse Lethality Assay (MLA) [33] in accordance with Italian and European legislation on ethical standards (European Communities Council Directive (2010/63/EU) on the protection of animals used for scientific purposes). The same test was also applied to the sera collected from the cardiac clot of the mallard ducks, the little grebe, and the little egret, while serum from the wood sandpipers were not tested due to the small quantity.

## 3. Results

Seven mallard ducks (*Anas platyrhynchos*), 4 common teals (*Anas crecca*), 1 garganey (*Anas querquedula*), 2 wood sandpipers (*Tringa glareola*), 1 little egret (*Egretta garzetta*), 1 little grebe (*Tachybaptus ruficollis*), and 4 Eurasian coots (*Fulica atra*) were found dead. The death of many other birds was reported from rangers employed in the natural area, but the number of victim animals was uncertain and probably underestimated given the wideness of the wetland. A mallard duck and a wood sandpiper were found still alive but unable to fly with flaccid paralysis of wings and neck; they died within a few hours.

No macroscopic lesions were observed except for the generalized congestion of the organs. Gastrointestinal tract was empty in ducks and in the little grebe, while small quantities of food material were found in the stomach of the wood sandpipers and little egret. Test for Newcastle Disease virus and Avian influenza virus from the brain samples gave negative results. Conversely, enriched broths previously inoculated with liver and intestinal samples resulted positive for *C. botulinum* type C/D by means of biomolecular techniques [26,32].

Sera from mallard ducks and the little egret were neutralized by C antitoxin, confirming the botulism diagnosis. On the contrary, serum from the little grebe gave negative results. This was presumably due to the different amounts of samples (0.4–0.5 mL and 0.2 mL, respectively) and, consequently, BoNTs. In fact, MLA is not very sensitive to low levels of BoNTs [34].

## 4. Discussion

Interestingly, the mosaic C/D type botulism was responsible for the outbreak observed in the wild birds of the reserve. Although several botulism outbreaks in wild species have been previously reported as type C botulism [25,35], it is possible that some of them could be misidentified as C type but were actually C/D type. In fact, MLA and the most of Polymerase Chain Reaction (PCR) assays described in literature and generally used to diagnose botulism fail to distinguish mosaic C/D type from non-mosaic C type [26]. For this reason, positive sera from mallard ducks were neutralized by C antitoxin. More recent diagnostic assays as macroarray methods highlighted the association between mosaic type C/D and botulism outbreak in wild birds of Europe [26,36]. Considering the finding of *C. botulinum* type C/D in faecal samples of waterbirds [37], migratory birds may have contributed to carrying the bacteria in the reserve, causing the outbreak when the suitable environmental conditions occurred.

Considering the increase of type C/D toxin production in presence of decomposing carcasses [16], we suggest that the raised mortality of fishes that occurred in the artificial pond could have played a relevant role by increasing the amount of decaying organic material in the environment, predisposing C/D toxin production.

Other factors in the reserve may have contributed to the production of the toxin. The average temperature registered when the outbreak occurred was 28–30 °C, that represents the optimal temperature for *C. botulinum* growth and toxin production [17]. This is a possible explanation of the increase of the botulism outbreaks in the summertime. Therefore, most of the outbreaks occur during the summer and fall [38].

Although fishes from tanks were not tested and fishes are usually reservoirs of *C. botulinum* type E [17], we cannot exclude that they have played a direct role in the outbreak, considering (i) that decayed fishes could promote favourable growth conditions for *C. botulinum* type C other than type E; (ii) the implication of tilapia (*Oreochromis mossambicus*) as the source of type C toxin in pelicans [39]; and (iii) the reports about type C botulism outbreaks involving a relevant number of pelicans, herons, and other fish-eating birds [24,40]. Carcass-maggots are generally considered a possible source of toxin for wild birds because they are unaffected by the toxin and can accumulate it [17], but they were not detected in the gastrointestinal tract of the birds examined in this study.

The prompt removal of the fish carcasses led to a rapid resolution of the outbreak of the disease in the waterfowl of the reserve. This highlights the importance of a correct management for any action in environmental contexts. In conclusion, any human activity in wildlife habitats should be carefully considered in order to assess the possible impacts and to quickly identify the possible risks of changes in wildlife.

## Figures and Tables

**Table 1:** List of waterfowl species of the reserve.

Audouin’s Gull (*Larus audouinii*)	Black Tern (*Chlidonias niger*)	Black-crowned Night Heron (*Nycticorax* *nycticorax*)
Black-headed Gull (*Larus ridibundus*)	Black-necked Grebe (*Podiceps* *nigricollis*)	Black-tailed Godwit (*Limosa limosa*)
Black-throated Loon (*Gavia* *arctica*)	Black-winged Stilt (*Himantopus himantopus*)	Common Crane (*Grus grus*)
Common Greenshank (*Tringa nebularia*)	Common Moorhen (*Gallinula chloropus*)	Common Pochard (*Aythya ferina*)
Common Redshank (*Tringa tetanus*)	Common Ringed Plover (*Charadrius hiaticula*)	Common Sandpiper (*Actitis hypoleucos*)
Common Shelduck (*Tadorna tadorna*)	Common Snipe (*Gallinago gallinago*)	Cory’s Shearwater (*Calonectris* *diomedea*)
Curlew Sandpiper (*Calidris ferruginea*)	Dunlin (*Calidris alpine*)	Eurasian Bittern (*Botaurus* *stellaris*)
Eurasian Coot (*Fulica atra*)	Eurasian Curlew (*Numenius arquata*)	Eurasian Oystercatcher (*Haematopus ostralegus*)
Eurasian Spoonbill (*Platalea* *leucorodia*)	Eurasian Teal (*Anas crecca*)	Eurasian Wigeon (*Anas Penelope*)
Eurasian Woodcock (*Scolopax rusticola*)	European Golden Plover (*Pluvialis apricaria*)	Ferruginous Duck (*Aythya nyroca*)
Gadwall (*Anas strepera*)	Garganey (*Anas Querquedula*)	Glossy Ibis (*Plegadis* *falcinellus*)
Great Cormorant (*Phalacrocorax* *carbo*)	Great Crested Grebe (*Podiceps* *cristatus*)	Green Sandpiper (*Trinca ochropus*)
Grey Heron (*Ardea* *cinerea*)	Grey Plover (*Pluvialis squatarola*)	Greylag Goose (*Anser* *anser*)
Jack Snipe (*Lymnocryptes minimus*)	Kentish Plover (*Charadrius alexandrines*)	Kingfisher (*Alcedo atthis*)
Lesser Black-backed Gull (*Larus fuscus*)	Little Bittern (*Ixobrichus* *minutus*)	Little Crake (*Porzana parva*)
Little Egret (*Egretta* *garzetta*)	Little Grebe (*Tachyhaptus* *ruficollis*)	Little Gull (*Larus minutus*)
Little Ringed Plover (*Charadrius dubius*)	Little Stint (*Calidris minuta*)	Little Tern (*Sternula albifrons*)
Long-tailed Jaeger (*Stercorarius longicaudus*)	Mallard (*Anas platyrhynchos*)	Mediterranean Gull (*Larus melanocephalus*)
Northern Gannet (*Morus* *bassanus*)	Northern Lapwing (*Vanellus vanellus*)	Northern Pintail (*Anas acuta*)
Northern Showeler (*Anas Clypeata)*	Pied Avocet (*Recurvirostra avosetta*)	Purple Heron (*Ardea* *purpurea*)
Red-breasted Merganser (*Mergus serrator*)	Ruddy Turnstone (*Arenaria interpress*)	Ruff (*Philomacus pugnax*)
Sandwich Tern (*Thalasseus sandvicensis*)	Spotted Crake (*Porzana porzana*)	Spotted Redshank (*Tringa erythropus*)
Squacco Heron (*Ardeola* *ralloides*)	Temminck’s Stint (*Calidris temminckii*)	Tufted Duck (*Aythya fuligula*)
Tundra Bean Goose (*Anser* *serristrosis*)	Water Rail (*Rallus acquaticus*)	Western Great Egret (*Ardea* *alba*)
Whiskered Tern (*Chlidonias hybrid*)	White-winged Tern (*Chlidonias leuctoperus*)	Wood Sandpiper (*Trinca glareola*)
Yelkouan Shearwater (*Puffinus* *yelkouan*)	Yellow-legged Gull (*Larus michahelis*)	
